# Molecular Chaperones as Targets to Circumvent the CFTR Defect in Cystic Fibrosis

**DOI:** 10.3389/fphar.2012.00137

**Published:** 2012-07-17

**Authors:** Rebecca A. Chanoux, Ronald C. Rubenstein

**Affiliations:** ^1^Division of Pulmonary Medicine and Cystic Fibrosis Center, The Children’s Hospital of PhiladelphiaPhiladelphia, PA, USA; ^2^Department of Pediatrics, Perelman School of Medicine at the University of PennsylvaniaPhiladelphia, PA, USA

**Keywords:** CFTR, chaperone, endoplasmic reticulum, ERAD, heat shock protein, phenylbutyrate

## Abstract

Cystic Fibrosis (CF) is the most common autosomal recessive lethal disorder among Caucasian populations. CF results from mutations and resulting dysfunction of the Cystic Fibrosis Transmembrane Conductance Regulator (CFTR). CFTR is a cyclic AMP-dependent chloride channel that is localized to the apical membrane in epithelial cells where it plays a key role in salt and water homeostasis. An intricate network of molecular chaperone proteins regulates CFTR’s proper maturation and trafficking to the apical membrane. Understanding and manipulation of this network may lead to therapeutics for CF in cases where mutant CFTR has aberrant trafficking.

## Introduction

The most common disease-causing mutation in cystic fibrosis transmembrane conductance regulator (CFTR) is the deletion of a single phenylalanine at position 508, ΔF508-CFTR. This mutation is present in one or both alleles of ~90% of people with CF (Riordan, 2008), making it an attractive target for therapeutics. In contrast to wild type CFTR, which reaches the apical cell surface after its N-linked oligosaccharides are modified in the Golgi to an endoglycosidase H digestion-resistant form, ΔF508-CFTR does not acquire endoglycosidase H resistance (Cheng et al., 1990). These data suggested that ΔF508-CFTR is retained in the endoplasmic reticulum (ER; Kerem et al., 1989; Collins, 1992; Riordan, 1999; Bobadilla et al., 2002). Interestingly, ΔF508-CFTR appears to retain some ability to transport chloride when in the ER (Pasyk and Foskett, 1995), suggesting that the deletion of phenylalanine interferes with proper biogenesis and promotes degradation of the mutant protein (Ward and Kopito, 1994; Ward et al., 1995; Okiyoneda et al., 2010).

Because ΔF508-CFTR retains the ability to transport chloride, it is widely hypothesized that correction of the mutant protein’s trafficking would lead to functional CFTR at the apical cell surface (Denning et al., 1992b; Li et al., 1993; Pasyk and Foskett, 1995). This premise was supported by early data from Drumm et al. (1991), indicating that ΔF508-CFTR was functional in *Xenopus* oocytes, which are typically incubated at room temperature. Studying mammalian cells, Denning et al. (1992a) found that decreasing the cell incubation temperature led to an increase in both expression and function of ΔF508-CFTR at the cell surface. Overcoming this kinetic trafficking defect of ΔF508-CFTR would be an important step in developing therapeutics for people with CF.

## CFTR Biogenesis

Proper biogenesis of the CFTR protein is not a trivial task. CFTR is synthesized as a ~140 kDa protein (comprising 1480 amino acids) and requires a number of processing steps to progress to a mature, ~180 kDa form. The protein contains two nucleotide binding domains (NBD1 and NBD2), two membrane-spanning domains (MSD1 and MSD2), and an intervening regulatory domain (R; Riordan et al., 1989). During translation, MSD1 is synthesized first, followed by NBD1, R, MSD2, and finally NBD2; folding of the nascent peptide appears to occurs both co-translationally and post-translationally (Du et al., 2005; Kleizen et al., 2005).

F508 is located in NBD1, and while the crystal structures of wild type and ΔF508 NBD1 are quite similar, deletion of F508 appears to cause NBD1 to have a more unfolded solution conformation, as assessed by proton-deuterium exchange (Lewis et al., 2005, 2010). Furthermore, deletion of F508 appears to destabilize a critical interaction of NBD1/MSD2 interaction (Thibodeau et al., 2005; Serohijos et al., 2008). Du et al. (2005) also suggested that phenylalanine 508 provides an important interaction with NBD2 that assists in proper post-translational folding of this domain. Together, these data suggest that newly synthesized ΔF508-CFTR is less appropriately folded, and therefore more readily recognized by ER quality control mechanisms and targeted for degradation.

Interestingly, Cui et al. (2007) found that a wild type CFTR construct lacking the NBD2 domain escaped degradation and trafficked to the cell membrane where it had similar stability to full-length CFTR, but had a very low open probability. These data suggest that, though important for CFTR activity, NBD2 is not essential for CFTR biogenesis and exit from the ER. Consistent with this notion, when this group introduced the ΔF508 mutation into their NBD2-deficient construct, the resulting protein did not reach the plasma membrane, supporting the earlier hypothesis that ΔF508 impacts aspects of CFTR folding and biogenesis other than the NBD1/NBD2 interaction.

## Molecular Chaperones

To better understand the difficulties of ΔF508-CFTR biogenesis, it is important to examine the cellular context in which CFTR biogenesis occurs. The folding and trafficking environment, referred to by Wang et al. (2006) as the “CFTR interactome,” contains over 200 proteins that co-immunoprecipitate with either wild type or ΔF508-CFTR in model systems. These co-precipitating proteins, a number of which are implicated in proper folding, trafficking, and function of CFTR, include a number of molecular chaperone proteins. Molecular chaperones are proteins that aid in the folding of other proteins, but do not become part of the final product (Ellis, 1987). Instead, they promote self-assembly of their client proteins and prevent non-productive folding. Historically, the functions of many molecular chaperones are defined by their ability to assist in the refolding of denatured proteins, such as luciferase, *in vitro* (Schroder et al., 1993; Barral et al., 2004).

Molecular chaperones appear to interact with CFTR during many stages of biogenesis. Nascent peptides of membrane proteins, such as CFTR, are synthesized at the ER, where co-translational folding occurs (Hartl, 1996). Because CFTR is inserted into the ER membrane, its folding is monitored by chaperone proteins within both the ER and cytoplasm. If CFTR folding is delayed or prolonged, interaction with molecular chaperones (Loo et al., 1998; Meacham et al., 1999) can cause improperly folded proteins to be transported back to the cytoplasm, where they are targeted for degradation by the proteasome (reviewed in Rivett, 1993). This process, known as ER-associated degradation (ERAD), also involves a number of molecular chaperones. These interactions and processes are discussed in detail below.

Appropriately folded CFTR exits the ER and is transported to the Golgi where its N-linked glycosyl modification is further processed into the mature form before trafficking to the apical cell surface. The ΔF508-CFTR mutant is unable to reach the Golgi, though it is able to transport chloride in reconstituted systems (Li et al., 1993; Lukacs et al., 1993). A number of data suggest differing and not mutually exclusive mechanisms by which ΔF508-CFTR is retained in the ER. One proposed mechanism suggests that recognition of an ER exit sequence within NBD1 of the CFTR protein by Coat Complex II (COP II) ER → Golgi transport machinery is impaired in the ΔF508 protein (Chang et al., 1999; Wang et al., 2004). Other works cite improper and/or more robust chaperone binding as the mechanism by which ΔF508-CFTR is retained in the ER (Pind et al., 1994; Wang et al., 2006). Hypothetically, excessive chaperone binding could inhibit COP II’s access to the ER exit motif within NBD1. To address this question, Wendeler et al. (2007) affixed a strong ER exit signal to the wild type CFTR protein. This signal did not disrupt protein localization or expression, but did enhance wild type CFTR maturation by two-fold. In contrast, this ER exit signal did not enhance the maturation of the ΔF508 protein, thereby contradicting the hypothesis that a primary defect in the ER exit sequence is responsible for failure in ΔF508-CFTR trafficking. Instead, these data support the hypothesis that molecular chaperone proteins may play a key role in the quality control of wild type CFTR.

## CFTR and ERAD

Accumulated non-functional membrane or ER luminal proteins can aggregate and interfere with the production or function of other newly synthesized proteins, as well as cause an ER and/or cellular stress response. To prevent this, aberrant proteins are recognized, shuttled out of the ER, and targeted for degradation by ERAD.

Ciechanover and colleagues demonstrated that Hsc70, the constitutively expressed 70 kDa heat shock protein, is required for the ubiquitin-directed proteasome-mediated degradation of a number of cellular proteins (Bercovich et al., 1997); this ubiquitin-proteasome pathway is also operative in ERAD. Hsc70 has a variety of roles in the cell, including uncoating clathrin-coated pits and promoting protein ubiquitination and both proteasomal and lysosomal degradation (Chiang et al., 1989; DeLuca-Flaherty et al., 1990; Bercovich et al., 1997; Morgan et al., 2001). Because improperly folded CFTR undergoes ubiquitination-mediated degradation (Jensen et al., 1995; Ward et al., 1995), it was hypothesized that Hsc70 promotes ERAD of ΔF508-CFTR. In fact, ΔF508-CFTR associates more robustly with Hsc70 than wild type CFTR (Strickland et al., 1997; Meacham et al., 1999; Rubenstein and Zeitlin, 2000). Furthermore, pharmacologic disruption of Hsc70 binding to either wild type or ΔF508-CFTR decreases CFTR ubiquitination (Fuller and Cuthbert, 2000), stabilizes the ER (immature band B) form of CFTR (Fuller and Cuthbert, 2000), and can promote CFTR maturation (Jiang et al., 1998).

**Figure 1:**
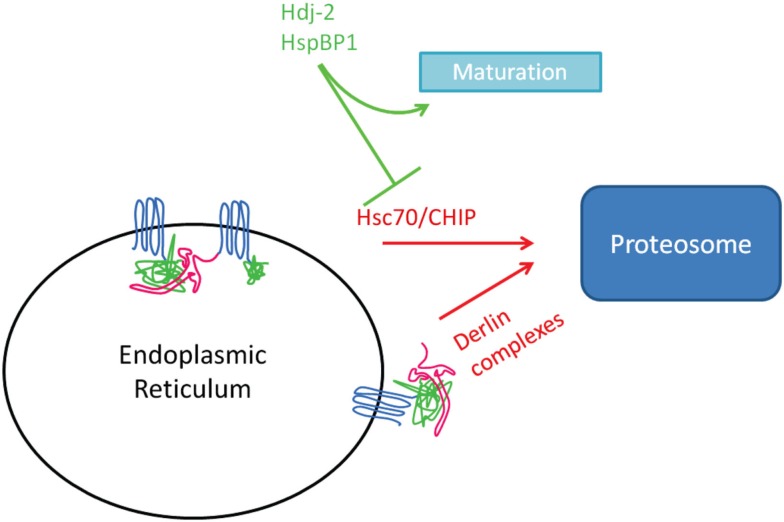
**Complexes implicated in ERAD**.

Investigations in our group have focused on the mechanism by which 4-phenylbutyrate (4PBA) enhances ΔF508-CFTR trafficking (Rubenstein et al., 1997). We found that 4PBA decreased Hsc70 mRNA and protein expression in CF epithelial cells, as well as decreased recovery of ΔF508-CFTR when Hsc70 was immunoprecipitated (Rubenstein and Zeitlin, 2000; Rubenstein and Lyons, 2001). These data support the hypothesis that Hsc70 inhibits ΔF508-CFTR maturation, likely by promoting its ERAD (see Figure 1).

Hsc70’s promotion of ERAD involves a co-chaperone known as CHIP (C-terminus of Hsc70-interacting protein), an E3 ubiquitin ligase (Wiederkehr et al., 2002; Murata et al., 2003). Meacham et al. (2001) demonstrated that CHIP and Hsc70 cooperate to target the immature (band B) form for ubiquitination and degradation; overexpression of CHIP decreased whole cell and surface expression of CFTR. Simplistically, association of Hsc70 with a client (like CFTR) would bring CHIP into proximity where it could catalyze ubiquitination of the client. A more robust association of Hsc70 with client, as was demonstrated by our group for ΔF508 vs. wild type CFTR (Rubenstein and Zeitlin, 2000), would portend greater ubiquitination and likelihood for ERAD.

Additional co-chaperone proteins interact with the Hsc70/CHIP complex to modulate their client interaction. HspBP1 binds Hsc70 and this binding decreases the ubiquitin ligase activity of CHIP (Alberti et al., 2004). This, in turn, decreases the ubiquitin-mediated degradation of CFTR and increased the steady-state expression of either wild type or ΔF508-CFTR in an *in vitro* assay. Similarly, Bag-2 interacts with CHIP and inhibits its ubiquitin ligase activity (Arndt et al., 2005). With regards to CFTR, increased Bag-2 expression increases steady-state expression of both immature and mature CFTR in heterologous cells (Arndt et al., 2005). Bag-2 appears to stabilize the NBD1 domain of CFTR and prevent its aggregation while unfolded. Matsumura et al. (2011) performed experiments in a cell-free system to discern the role of Hsc70 in promoting biogenesis from its role in promoting ubiquitination. Using a fragment of the Bag-1 protein to destabilize the interaction between Hsc70 and CFTR led to a decrease in CFTR ubiquitination, but no effect on protein biogenesis (Matsumura et al., 2011). Similarly, Meacham et al. (1999) found that the interaction between Hsc70 and Hdj-2 promotes stabilization of a folding-competent CFTR intermediate and prevents aggregation of NBD1, while Zhang et al. (2006) also found that Hdj-2/Hsc70 promoted stabilization of mature CFTR and prevented aggregation. Together, these data suggest that Hsc70 and CHIP primarily cooperate to promote ERAD of clients, and that this interaction can be modified by co-chaperones. In the case of ΔF508-CFTR, a more robust association with Hsc70/CHIP portents increased ERAD.

In addition to Hsc70, degradation of newly synthesized ΔF508-CFTR is also controlled by Derlin, an ER membrane-associated complex comprised of RMA1 (an E3 ubiquitin ligase), Ubc6e (an E2 ubiquitin-conjugating enzyme), and Derlin-1 (Younger et al., 2006). Derlin-1 appears to retain ΔF508-CFTR at the ER membrane and allow its recognition by Ubc6e and RMA1. Derlin-1 can interact with p97, the ATPase that extracts proteins from the ER during ERAD, within a separate complex that also targets CFTR for degradation (Sun et al., 2006). Derlin-1 overexpression leads to decreased wild type and ΔF508-CFTR expression, while RNAi-mediated depletion of Derlin-1 had the opposite effect. Interestingly, the Derlin complex can ubiquitinate proteins co-translationally (Younger et al., 2006), which is known to occur for CFTR (Sato et al., 1998) while CHIP/Hsc70 primarily recognizes misfolded proteins post-translationally (Younger et al., 2006). Derlin-1 degrades the CFTR fragment containing only MSD1, but not longer forms of the protein, possibly because partial CFTR folding prevents binding of Derlin-1 (Sun et al., 2006). Together, these data suggest that Derlin and CHIP/Hsc70 have complementary roles in surveillance of newly synthesized proteins to prevent accumulation of misfolded proteins.

## CFTR and Chaperones in the Cytoplasm

Folding of the cytosolic domains of CFTR requires coordinated effort of heat shock proteins (Hsps), a large family of functionally related chaperones that promote folding and prevent aggregation of new proteins. ΔF508-CFTR demonstrates prolonged interaction with cytosolic Hsps (Yang et al., 1993; Loo et al., 1998; Rubenstein and Zeitlin, 2000; Choo-Kang and Zeitlin, 2001), indicating that these chaperones also represent potential therapeutic targets in improving ΔF508-CFTR trafficking.

Hsp70, the stress induced 70 kDa heat shock protein, and the aforementioned Hsc70, are two extensively studied members of this family. They are more than 85% identical on an amino acid level, which has led many to hypothesize that Hsp70 and Hsc70 have similar functions. Interestingly, however, Hsp70 function does not always overlap with Hsc70’s, and the two often have opposite cellular effects (Gething and Sambrook, 1992; Goldfarb et al., 2006). Experimentally, Hsc70 inhibition has been shown to lead to an increase in Hsp70 expression (Aquino et al., 1996); this may represent cellular stress, as Hsp70 expression is induced by such stress (reviewed in Mayer and Bukau, 2005).

**Figure 2:**
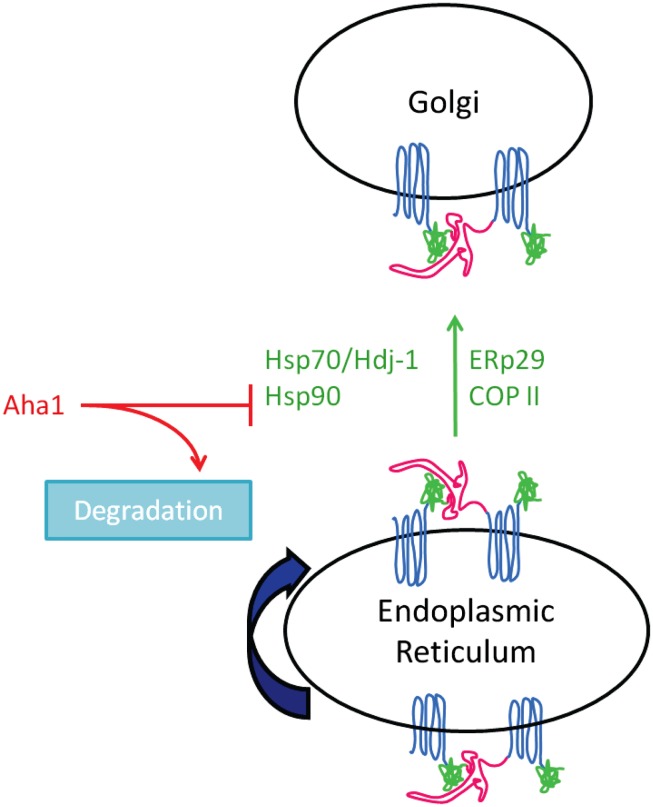
**Chaperone proteins implicated in ΔF50S-CFTR biogenesis and maturation**.

The exact role of Hsp70 in CFTR function and expression remains controversial. Choo-Kang and Zeitlin examined the effect of increased Hsp70 expression on CFTR in CF epithelial cells. In contrast to previous data (Rubenstein and Zeitlin, 2000), their data suggested that 4PBA increased Hsp70 expression and increased Hsp70/CFTR interaction (Choo-Kang and Zeitlin, 2001). They also found that overexpression of Hsp70 enhanced the interaction between Hsp70 and ΔF508-CFTR, which promoted ΔF508-CFTR maturation (see Figure 2). Suaud et al. (2011b) recently reconciled these data and demonstrated that 4PBA causes a transient increase in Hsp70 expression by a mechanism that involves the STAT-3 transcription factor and its interacting protein, Elongator Protein 2 (Elp2). This transient increase in Hsp70 expression with 4PBA is consistent with that suggested by gene expression profiling experiments (Wright et al., 2004). Taken together, these data support a model in which Hsp70 promotes proper trafficking of ΔF508-CFTR; this contrasts the role of its homolog, Hsc70, which, as discussed above, appears to promote ΔF508-CFTR degradation by ERAD.

In contrast, Farinha et al. (2002) found no increase in either wild type or ΔF508-CFTR maturation when both CFTR and Hsp70 were overexpressed in Chinese Hamster Ovary (CHO) cells. Instead, they saw increased wild type CFTR maturation only when Hsp70’s co-chaperone, Hdj-1, was also overexpressed, but did not see a similar increase in maturation of ΔF508-CFTR. They found that Hsp70/Hdj-1 could slow the degradation rate of wild type CFTR, but not the mutant protein, possibly because of the folded state of ΔF508-CFTR. Farinha et al. also examined 4PBA treatment of cells to determine if the effect was similar to the results of their transient Hsp70/Hdj-1 overexpression. They observed a more rapid degradation of ΔF508-CFTR with 4PBA treatment, but no effect on wild type CFTR. This is contradictory to what was seen in previous reports, which suggest 4PBA promotes ΔF508-CFTR trafficking (Rubenstein et al., 1997; Choo-Kang and Zeitlin, 2001; Suaud et al., 2011b). This apparent disparity may result from the model systems under study. Farinha et al. used heterologous CHO cells where CFTR (wild type or ΔF508) was overexpressed, while others (Rubenstein et al., 1997; Choo-Kang and Zeitlin, 2001; Suaud et al., 2011b) used IB3-1 CF bronchiolar epithelial cells where ΔF508-CFTR is endogenously expressed.

Another heat shock protein, Hsp90, also plays a key role in protein homeostasis and folding of a variety of proteins in a number of organisms (reviewed in Balch et al., 2008; Hutt et al., 2009; Powers et al., 2009). CFTR folding intermediates are stabilized by binding to Hsp90, which prolongs their half-life and aids in their trafficking and maturation (Loo et al., 1998; Fuller and Cuthbert, 2000; Wang et al., 2006). Hsp90 binding to client depends on its ATPase activity, and both client binding and Hsp90 ATPase activity are enhanced by the presence of co-chaperones, such as Aha1 (Pearl and Prodromou, 2006). Recently, Aha1 was suggested to regulate CFTR interaction with Hsp90, leading to increased interest in this co-chaperone (Wang et al., 2006). Sun et al. (2008) examined chaperone binding of wild type and ΔF508-CFTR and found that both proteins interacted similarly with Hsp90. Interestingly, they found that Aha1 interacted with ΔF508-CFTR at almost twice the affinity of wild type CFTR (Sun et al., 2008). They also expressed CFTR fragments in an attempt to rescue ΔF508-CFTR trafficking, as was reported in previous studies (Owsianik et al., 2003; Clarke et al., 2004; Cormet-Boyaka et al., 2004). With one such fragment of CFTR, they saw the predicted increase in ΔF508-CFTR maturation and a corresponding decrease in Aha1 binding to ΔF508-CFTR. These data suggest that Aha1 plays an important role in the Hsp90-mediated stabilization of CFTR. Koulov et al. (2010) recently extended these findings by demonstrating that mutations introduced in both the N- and C-terminal structures of Aha1 decreased binding of Aha1 to Hsp90, which in turn decreased the ATPase activity of Hsp90 and its ability to bind client proteins. Taken together, these data suggest that Aha1 promotes the binding of Hsp90 to client proteins by increasing the Hsp90’s ATPase activity.

While initial studies using Hsp90 inhibitors, such as geldanamycin, suggested that Hsp90 promotes ΔF508-CFTR maturation and trafficking (Loo et al., 1998; Wegele et al., 2004), studies focused on Hsp90 and Aha1 suggest an alternate mechanism (Wang et al., 2006; Koulov et al., 2010). It is likely that, similar to Hsc70, the Hsp90/CFTR interaction is complex. Perhaps initial binding between Hsp90 and CFTR lead to productive biogenesis. However, if the interaction is prolonged by CFTR’s inability to fold, CFTR is targeted for degradation instead.

While many studies focus on correcting the trafficking of ΔF508-CFTR to the apical cell surface, there is evidence that regulation of this mutant’s endocytic trafficking is also abnormal. In fact, wild type CFTR is efficiently recycled back to the apical cell membrane after endocytosis. In contrast, ΔF508-CFTR that is delivered to the membrane using low temperature is removed from the surface more rapidly and is recycled less efficiently than the wild type CFTR (Cholon et al., 2009). These data suggest that increasing the fraction of ΔF508-CFTR that arrives at the apical cell surface, while important, may not be sufficient to increase the functional expression of this mutant protein. Interestingly, because Hsc70 is involved in endocytosis and the uncoating of clathrin-coated vesicles (DeLuca-Flaherty et al., 1990; Morgan et al., 2001), and for targeting proteins for degradation by the lysosomes (Gething and Sambrook, 1992), it seems likely that Hsc70 may also influence the stability of the wild type and mutant CFTR proteins that are expressed on the apical cell surface. These data also suggest that therapeutics which modulate the effect of Hsc70 on clathrin-mediated endocytosis may lead to increased apical membrane stability of ΔF508-CFTR.

## CFTR and Chaperones in the Endoplasmic Reticulum

The role of ER luminal chaperones in CFTR biogenesis is less well delineated. CFTR biogenesis appears influenced by additional molecular chaperone proteins in the ER, including calreticulin and calnexin. These proteins recognize terminal oligosaccharides on proteins modified with high mannose N-linked glycosylation and promote ER retention of “folding intermediates” until they either fold properly or undergo ERAD. As such, Harada et al. (2006, 2007) found that CFTR expression and function were enhanced by RNAi-mediated depletion of calreticulin in both cultured cells and mouse models, suggesting that calreticulin negatively regulates CFTR. Because previous reports indicated that curcumin, a SERCA pump inhibitor, corrected ΔF508-CFTR trafficking to the apical plasma membrane (Egan et al., 2004), Harada et al. (2007) examined the mechanism by which this occurs. They found that curcumin downregulates calreticulin expression, leading to enhanced CFTR expression. Though curcumin alone could not activate ΔF508-CFTR in their experiments, in combination with calreticulin knockdown they showed enhanced activity of mutant CFTR, again consistent with calreticulin negatively regulating CFTR.

Calnexin’s role in regulating CFTR biogenesis is less clear. Initial reports suggest that calnexin binds to immature CFTR, and the interaction with ΔF508-CFTR is prolonged, compared to wild type CFTR (Pind et al., 1994). Based on these data, it is reasonable to hypothesize that calnexin is responsible for ER retention of ΔF508-CFTR, and may therefore represent a viable target for therapeutics to rescue ΔF508-CFTR. However, recent studies suggest a more complex picture of CFTR regulation by calnexin. One study suggested that calnexin actually decreased ERAD of ΔF508-CFTR (Okiyoneda et al., 2004), and depletion of calnexin using RNAi did not improve trafficking of newly synthesized ΔF508-CFTR (Farinha and Amaral, 2005). While calnexin might not influence CFTR trafficking as predicted, this study may have been limited by incomplete calnexin depletion. To address this possibility, a follow-up study examined CFTR trafficking in calnexin-deficient cells, or cells containing calnexin mutant proteins (Okiyoneda et al., 2008). One calnexin mutant, a truncated form that is exported from the ER, was able to bind to ΔF508-CFTR with similar affinity to wild type. However, this mutant failed to increase the amount of ΔF508-CFTR in the Golgi, suggesting that calnexin may not be responsible for ER retention of ΔF508-CFTR. In complimentary experiments, the group also employed wild type and calnexin knockout murine embryonic fibroblasts (MEFs) to address caveats of earlier RNAi experiments. They found that wild type CFTR protein was decreased in calnexin knockout MEFs, compared to MEFs containing wild type calnexin. Consistent with the RNAi experiments, they found that neither ΔF508-CFTR trafficking nor chloride transport was affected by calnexin knockout. These data suggest that calnexin is not sufficient for ER retention and degradation of the ΔF508-CFTR protein. Instead, other ER chaperone proteins may represent a stronger therapeutic target for CF patients.

Endoplasmic reticulum luminal chaperones involved in the unfolded protein response (UPR) work closely with the ERAD system. When protein folding in the ER is delayed, the UPR is activated to reestablish homeostasis within the ER by increasing the protein folding capacity of the cell and/or decreasing biosynthesis (reviewed in Schroder and Kaufman, 2005). The UPR is comprised of the regulator protein Grp78/BiP and a number of signal transducers, including ATF6 and PERK (Bertolotti et al., 2000; Lee, 2005). Under non-stress conditions, Grp78/BiP binds ATF6 and maintains it in an inactive state. Under ER stress, such as an excess of unfolded protein, Grp78/BiP preferentially binds to the luminal unfolded protein, which releases and allows activation of ATF6 and PERK, leading to initiation of the UPR.

Because ΔF508-CFTR is a misfolded protein, Kerbiriou et al. hypothesized that ΔF508-CFTR-expressing cells would activate the UPR. Using ATF6 and Grp78/BiP as markers of the UPR, they found that protein levels of both Grp78/BiP and activated ATF6 were increased in ΔF508-CFTR-containing cells (Kerbiriou et al., 2007). Interestingly, RNAi-mediated depletion of ATF6, but not Grp78/BiP, corrected ΔF508-CFTR trafficking, as evidenced by increased ΔF508-CFTR-mediated chloride transport and surface expression. These data suggest that the UPR pathway is involved in the retention of ΔF508-CFTR in the ER, but that Grp78/BiP is not involved directly in CFTR biogenesis. This is also consistent with earlier data from Yang et al. (1993) and Pind et al. (1994), which found no interaction between CFTR and Grp78/BiP, and no effect of Grp78/BiP on the trafficking of ΔF508-CFTR. In contrast to Kerbiriou et al. others have not found increased Grp78/BiP expression in cells expressing ΔF508-CFTR (Nanua et al., 2006). These seemingly contradictory findings may indicate a potentially transient interaction between unfolded proteins and Grp78/BiP. In addition, ERAD may be the predominant mechanism by which the cell responds to unfolded CFTR, meaning that Grp78/BiP’s role in the response to ΔF508-CFTR is small, leading to a small or negligible activation of the UPR. Based on these data, it remains unclear what role the UPR plays in trafficking or internal retention of ΔF508-CFTR.

Our group has recently focused on another ER chaperone and its potential role in regulating CFTR trafficking. ERp29 (ER luminal protein of 29 kDa) is ubiquitously expressed, but is especially prominent in brain and lung (Demmer et al., 1997). Its function is not entirely clear, but is suggested to promote thyroglobulin secretion and regulate assembly of connexin hemichannels (Sargsyan et al., 2002; Hubbard et al., 2004; Baryshev et al., 2006; Das et al., 2009), and it also seems to play a role in CFTR trafficking. Our group recently demonstrated that 4PBA increased ERp29 mRNA and protein expression (Suaud et al., 2011a). We also demonstrated that overexpression of ERp29 in *Xenopus* oocytes and mammalian cells increased the functional and surface expression of wild type and ΔF508-CFTR, while RNAi-mediated depletion of ERp29 decreased wild type CFTR in bronchial epithelial cells (Suaud et al., 2011a). These data suggested that ERp29 protein acts to promote biogenesis of both ΔF508 and wild type CFTR, and is the first ER luminal protein described to have this role. While additional studies are necessary, these data suggest an additional mechanism by which 4PBA may correct ΔF508-CFTR biogenesis and trafficking.

## Molecular Chaperones as Pharmacologic Targets

To improve the function of ΔF508-CFTR, it is important to consider the many molecular chaperones in the CFTR “interactome” as potential therapeutic targets. Though 4PBA is a prototype ΔF508-CFTR corrector, its effects are only partial. While most reports suggest that 4PBA promotes ΔF508-CFTR trafficking by decreasing Hsc70 and increasing Hsp70 (Rubenstein et al., 1997; Rubenstein and Zeitlin, 1998, 2000; Choo-Kang and Zeitlin, 2001; Rubenstein and Lyons, 2001; Suaud et al., 2011b), another found no 4PBA effect on these chaperones or on ΔF508-CFTR (Farinha et al., 2002). Early phase clinical trials showed a partial improvement in CFTR-mediated chloride transport in ΔF508-CFTR homozygous subjects with CF (Rubenstein and Zeitlin, 1998; Zeitlin et al., 2002), but the amount of improvement suggested that more efficacious correctors would be necessary to achieve meaningful clinical improvements.

**Figure 3:**
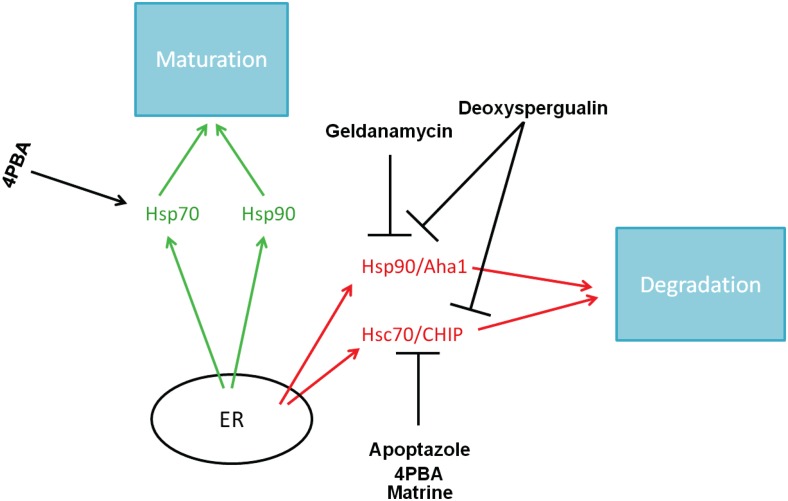
**AF508-CFTR correctors and their molecular targets**.

In addition to 4PBA, a variety of Hsc70 inhibitors are being examined as potential correctors of ΔF508-CFTR trafficking and may also represent therapeutic targets for treatment of CF (see Figure 3). Apoptazole is one such drug that interferes with Hsc70. Cho et al. (2011) found that apoptazole has the potential to promote ΔF508-CFTR trafficking and activity. Apoptazole appears to disrupt the ATPase activity of Hsc70 and decreases the ubiquitination of ΔF508-CFTR by blocking the interaction between Hsc70 and CHIP.

Matrine, a quinolizidine alkaloid, also downregulates Hsc70 expression, leading to an increase in ΔF508-CFTR protein levels (Basile et al., 2012). It also allows ΔF508-CFTR to exit the ER and localize to the plasma membrane, as evidenced by an increase in interaction between ΔF508-CFTR and BAG3, a co-chaperone located at the apical cell surface.

Deoxyspergualin is a drug that targets both Hsc70 and Hsp90 (Nadler et al., 1992; Nadeau et al., 1994), but has no apparent effect on Hsp70. Jiang et al. (1998) found that deoxyspergualin treatment increased CFTR activity in ΔF508-CFTR-expressing cells, suggesting this drug may provide an alternate mechanism by which to affect Hsc70 and indirectly increase ΔF508-CFTR trafficking. Clinically, there are many potential problems with deoxyspergualin treatment, however, likely because Hsc70 and Hsp90 are ubiquitously expressed proteins with many functions. Recently, Norez et al. explored a potential solution to this problem by constructing a form of the molecule with an adjuvant. When they generated a human serum albumin/deoxyspergualin construct, they were able to deliver the drug at lower doses, with lower toxicity, and achieve even better correction of ΔF508-CFTR trafficking than they saw with deoxyspergualin alone (Norez et al., 2008). This is a promising method by which drugs could be delivered to patients with lower toxicity.

Pharmacologic agents that specifically target Hsp90 are also being studied to understand their effects on ΔF508-CFTR. Early studies showed that geldanamycin, as well as other members of the ansamycin family, target Hsp90, and disrupt binding to CFTR (Loo et al., 1998). However, geldanamycin increased turnover of CFTR by increasing CFTR’s susceptibility to ERAD. Based on these data, it seems that geldanamycin would be detrimental, rather than helpful, in CF patients. However, more recent data provided a completely different picture. Using an *in vitro* system, Fuller and Cuthbert (2000) found that geldanamycin interferes with degradation of ΔF508-CFTR by disrupting ubiquitination. The caveat of this study is that it was conducted using rabbit reticulocyte lysates, rather than cell or animal models. Further investigation into geldanamycin or other Hsp90 inhibitors is needed and would provide a more complete picture of the role that these agents play in maturation of the mutant CFTR protein.

The identification of ER luminal chaperones, such as ERp29, that modulate CFTR and ΔF508-CFTR biogenesis is an exciting new development. These chaperones may be useful targets for development of novel ΔF508-CFTR corrector strategies.

## Conclusion

Patients currently receive therapeutics primarily aimed at treating symptoms of Cystic Fibrosis (CF; Ashlock and Olson, 2011; Cuthbert, 2011), although the first mechanism-based therapy for CF patients harboring a CFTR gating mutation like G551D was recently approved. For most people with CF this is not a permanent solution, thus new therapies that can target the underlying pathology of the defect are needed. This is a difficult task, as ΔF508-CFTR correctors tested thus far have had only limited efficacy (Rubenstein and Zeitlin, 1998), likely due to the complexities of CFTR folding and trafficking. Targeting chaperone proteins that influence CFTR, rather than CFTR itself holds promise for success. Because of their ubiquitous expression and interactions with so many cellular proteins, small changes in chaperone level or function may have dramatic effects on client proteins, such as CFTR.

It is important to keep in mind that the molecular chaperone functions described here (ERAD, UPR, folding, etc.) are tightly regulated and highly evolved to prevent the prolonged existence of unfolded or improperly folded proteins. In order to overcome the ΔF508-CFTR trafficking defect, it is necessary to find ways to bypass and/or change the set point of these quality control mechanisms. The system redundancy, highlighted by chaperone proteins with similar or overlapping roles (e.g., Hsc70/CHIP and Derlin), adds a level of security which is essential to the cell, but difficult to overcome, from a scientific perspective. A very delicate balance must be struck if a highly efficient therapeutic agent is to be found. The compound must prolong the lifetime of the misfolded ΔF508-CFTR protein, in order to allow proper folding. However, increased half-life might also lead to increased chaperone binding, which, as in the case of Hsp90, can counter-productively force the cell to degrade misfolded proteins (Koulov et al., 2010).

Because a large fraction of newly synthesized ΔF508-CFTR is degraded by the ubiquitin-proteasome pathway, inhibition of the proteasome inhibitors might seem like an attractive therapeutic strategy. However, inhibiting proteasomal degradation does not increase the functional ΔF508-CFTR at the apical cell surface (Ward and Kopito, 1994; Ward et al., 1995). Instead, inhibiting the proteasome led to intracellular accumulation of ubiquitinated immature ΔF508-CFTR without increasing surface expression and function. In addition, proteasomal inhibition leads to increased cellular stress due to accumulation of misfolded proteins, which in turn induces expression of heat shock proteins, such as Hsp70, Hsc70, and Hsp90 (Liao et al., 2006), and may lead to cell apoptosis/death (Fribley et al., 2004; Park et al., 2011). These data suggest that inhibition of the proteasome is not a viable therapeutic option for correcting ΔF508-CFTR trafficking.

Unfortunately, there are a number of difficulties that scientists face in designing therapeutics to correct ΔF508-CFTR. Many of the studies on CFTR and chaperones have been conducted using overexpression systems. This, of course, is necessary for detection of the extremely low-level expression of ΔF508-CFTR in cells where the protein is not overexpressed. However, this overexpression makes interpretation of the results somewhat more difficult. In addition, while often used non-epithelial cell models facilitate the overexpression of wild type and ΔF508-CFTR, non-epithelial cells do not endogenously express CFTR, so their responses to overexpression my not be physiologically relevant (as discussed above, Farinha et al., 2002). Studies performed in these models must be validated using epithelial cells.

CFTR expression varies between epithelial tissue types. Kalin et al. examined samples from CF patients as well as healthy human samples using immunohistochemistry. They found that the wild type CFTR protein could be detected in sweat glands, lung epithelia, and villi and goblet cells in the intestine (Kalin et al., 1999). In contrast, ΔF508-CFTR could not be detected in sweat glands, but expression in the lung and intestine were very similar to wild type CFTR. While this study did not address the functional activity of ΔF508-CFTR in these tissues, these data suggest that CFTR processing defects may be tissue type-specific and that ΔF508-CFTR processing may affect some tissues more than others. Further study of chaperone function in a range of epithelial tissues is required to fully understand their role in CFTR trafficking and activity.

Recent generation of novel animal models of CF, such as the ferret and pig, and their disease pathology is of great benefit to the advancement of this field as a whole (reviewed in Fisher et al., 2011) and (Keiser and Engelhardt, 2011). While the role of chaperones in CFTR trafficking have yet to be investigated in these models, future interrogations of epithelial cells from these models will undoubtedly yield a great deal of insights into both underlying physiology and therapeutic approaches.

Many chaperone proteins are upregulated in response to cellular stress, which may result from overexpression of exogenous proteins or increased abundance of misfolded proteins in the ER. Overexpressing ΔF508-CFTR may lead to a specific activation of proteins needed to fold the mutant, or instead cause a global upregulation of chaperone proteins involved in ERAD or the UPR, simply by increasing cellular stress. Studies examining overexpression of both wild type and ΔF508-CFTR lend credence to the hypothesis that the response is specific to the mutant protein, but this is still a concern that needs to be addressed when designing therapeutics.

Many pharmacologic agents that correct ΔF508-CFTR trafficking do so by an as yet unknown mechanism. Though many chaperones have been extensively studied, there are still aspects of our understanding that are lacking. This is evidenced by studies with seemingly contradictory data, discussed above. As an additional caveat, chaperone proteins have many targets and interact with an abundance of proteins in response to cellular stress. While changes in chaperone expression may positively influence ΔF508-CFTR expression, the effects on other important protein pathways could have unforeseen negative consequences. The use of these pharmacologic agents must be understood in the context of these other roles for chaperones within the cell. Building an even greater knowledge base of molecular chaperones and ΔF508-CFTR, in the context of the CFTR “interactome,” will help to fill in the gaps and lead to a better understanding of the pharmacologic agents, as well as the proteins that they target.

Finally, ΔF508-CFTR interacts with many other proteins during its lifetime, and it may not be possible to design a single molecule to correct all its potentially problematic interactions. Instead, a combination of therapeutics may be more appropriate and effective. Targeting multiple chaperones may allow therapies to avoid the trap of decreasing a single molecular chaperone protein too much. Small changes in multiple chaperones may provide the balance needed to prolong the life of ΔF508-CFTR enough to allow proper folding, but not so much that it is recognized by ERAD or the UPR. These sorts of small changes to multiple chaperones may also help create therapies with less toxic side effects.

## Conflict of Interest Statement

The authors declare that the research was conducted in the absence of any commercial or financial relationships that could be construed as a potential conflict of interest.
